# Breaking bad habits by improving executive function in individuals with obesity

**DOI:** 10.1186/s12889-018-5392-y

**Published:** 2018-04-16

**Authors:** Vanessa Allom, Barbara Mullan, Evelyn Smith, Phillipa Hay, Jayanthi Raman

**Affiliations:** 1 Health Psychology and Behavioral Medicine Research Group, School of Psychology and Speech Pathology, Curtin University, Perth, Australia; 2 Eating disorders and Obesity Psychology Research Clinic (EDOC), School of Social Sciences and Psychology, Western Sydney University, Penrith, NSW 2751 Australia; 3 Clinical and Health Psychology Research Initiative, School of Social Sciences and Psychology, Western Sydney University, Sydney, Australia; 4 School of Medicine, Western Sydney University, Sydney, Australia; 5 Clinical Psychology, Graduate School of Health, University of Technology Sydney (UTS), Sydney, Australia

**Keywords:** Obesity, Executive function, Habit, Weight loss intervention, Sedentary

## Abstract

**Background:**

Two primary factors that contribute to obesity are unhealthy eating and sedentary behavior. These behaviors are particularly difficult to change in the long-term because they are often enacted habitually. Cognitive Remediation Therapy has been modified and applied to the treatment of obesity (CRT-O) with preliminary results of a randomized controlled trial demonstrating significant weight loss and improvements in executive function. The objective of this study was to conduct a secondary data analysis of the CRT-O trial to evaluate whether CRT-O reduces unhealthy habits that contribute to obesity via improvements in executive function.

**Method:**

Eighty participants with obesity were randomized to CRT-O or control. Measures of executive function (Wisconsin Card Sort Task and Trail Making Task) and unhealthy eating and sedentary behavior habits were administered at baseline, post-intervention and at 3 month follow-up.

**Results:**

Participants receiving CRT-O demonstrated improvements in both measures of executive function and reductions in both unhealthy habit outcomes compared to control. Mediation analyses revealed that change in one element of executive function performance (Wisconsin Card Sort Task perseverance errors) mediated the effect of CRT-O on changes in both habit outcomes.

**Conclusion:**

These results suggest that the effectiveness of CRT-O may result from the disruption of unhealthy habits made possible by improvements in executive function. In particular, it appears that cognitive flexibility, as measured by the Wisconsin Card Sort task, is a key mechanism in this process. Improving cognitive flexibility may enable individuals to capitalise on interruptions in unhealthy habits by adjusting their behavior in line with their weight loss goals rather than persisting with an unhealthy choice.

**Trial registration:**

The RCT was registered with the Australian New Zealand Registry of Clinical Trials (trial id: ACTRN12613000537752).

## Background

Obesity is one of the leading risk factors of non-communicable diseases worldwide [49]. The prevalence of obesity remains high in developed nations and is increasing worldwide [34]. While some interventions to lose weight have been successful they are rarely successful at allowing people to maintain this weight loss in the long term [9, 23], where maintenance is clinically defined at least 1 year post weight loss [21]. The primary factors that contribute to obesity are unhealthy eating and sedentary behavior [49]. These behaviors are particularly difficult to change in the long-term because they are often enacted habitually [14, 40]. Moreover, weight loss maintenance will not be achieved if an intervention only temporarily interrupts these habits, as old behavioral patterns will resume once the intervention has ceased [13]. Therefore, there is a need to develop weight loss interventions that specifically target and aim to break the unhealthy habits that contribute to the maintenance of obesity.

A habit is an overlearned process that generates automatic responses to contextual cues [13], and is reinforced by repetition [27]. For example, arriving home after work each day and immediately having an unhealthy snack would likely lead to the formation of an unhealthy eating behavior habit such that a person may find themselves approaching the kitchen every time they enter the home regardless of hunger or desire to eat. The role of habit in unhealthy eating behavior and sedentary behavior has been demonstrated previously (for review, see: [12, 46]). Several studies have demonstrated that the more habitual a person’s eating and/or sedentary behavior is, the more likely they are to engage in that behavior [2, 8, 47]. Moreover, engaging in these behaviors leads to weight gain in the long term [32].

The few dietary interventions that have attempted to break habitual unhealthy eating behavior have shown promise in terms of maintenance of change in unhealthy eating or weight loss maintenance [7, 13]. For example, Carels et al. [7] compared the efficacy of a cognitive-based weight loss intervention with a habit-based intervention and found that while both approaches resulted in weight loss, the habit-based intervention resulted in weight-loss maintenance. The habit-based intervention was designed to disrupt unhealthy habits, and to develop new healthy habits. Further, preliminary results indicate that habit-based interventions are also successful at changing sedentary behavior [28]. For example, Matei et al. [28] demonstrated that prompting participants to replace sitting with light-intensity activity, and repeating and monitoring this action, led to a decrease in sedentary behavior and habits, and an increase in walking and activity habits.

In addition to unhealthy habits, poor executive function has been shown to be associated with unhealthy eating behavior [3], physical activity [19], and weight gain [33]. In particular, individuals with obesity have lower levels of executive function than their healthy weight counterparts, even after controlling for medical comorbidities [42]. Executive function refers to a set of higher order cognitive processes that includes planning, inhibiting behavior, cognitive flexibility, working memory and central coherence [5, 31]. Thus, executive function processes are essential to an individual’s ability to modify their behavior (for review, see: [1]). Researchers have demonstrated that improving elements of executive function results in reduction of unhealthy behaviors such as consumption of high caloric foods in healthy weight populations [4]. It might also be possible to change unhealthy habits that prevent weight loss in individuals with obesity by improving executive function. Dual-process theory of self-regulation [43] suggests that automatic, bottom-up processes, such as those that characterise habitual action, can be regulated by executive function to allow for top-down processes, such as goals, to direct behavior [20].

A manualised Cognitive Remediation Therapy (CRT) intervention program for adult obesity has been developed to improve executive function in individuals with obesity, targeting specifically those processes that individuals with obesity have difficulties with [36]. CRT, in particular, promotes reflection on thinking styles, develops metacognition and helps to explore and apply new thinking strategies in everyday life [44]. The primary function of CRT is to improve the thinking process rather than the content [45]. Findings from a recent randomized controlled trial in which adults with obesity underwent CRT for obesity (CRT-O) showed that those in the CRT-O group demonstrated significant improvement in executive function and an 6.6% average weight loss at the 3 months post treatment compared to a no-treatment control group [36, 37].

Given that improving executive function may also have an impact on habitual unhealthy behavior, the aim of the present study was to perform a secondary analysis of the CRT-O trial by Raman et al. [37] in order to determine if CRT-O decreases unhealthy eating and sedentary behavior habits, and if these changes are maintained at follow-up. An additional aim was to determine whether changes in executive function mediate any observed changes in habitual behavior. It is hypothesised that a reduction in both unhealthy eating and sedentary behavior habits will be observed after the intervention period in the CRT-O condition only and that these changes will be maintained at follow-up. Further, it is expected that the reduction in unhealthy habit strength observed in those who received CRT-O will be mediated by improvement in executive function.

## Methods

### Participants

Eighty participants with obesity, who had a body mass index (BMI) ranging between 30.12 kg/m^2^ and 60.23 kg/m^2^, *M* = 39.76; *SD* = 7.53, were recruited from the community via advertisements on social media sites, university and community centre notice boards and via media interviews with journalists from metropolitan and community newspapers. Participants ranged in age from 18 to 55 years, *M* = 41.39; *SD* = 7.85, and the majority of participants were female, *n* = 69. The inclusion and exclusion criteria have been outlined elsewhere [36].

### Measures

#### Executive function

A computerised version of the Wisconsin Card Sorting Test (WCST; [15, 16, 18]) was used as one measure of executive function. In this test, participants were presented with a deck of cards to sort according to one of three properties (colour, shape or number of shapes on them). Participants inferred which property by which to sort the cards based on feedback that indicated whether they were correct or not. After a string of correct responses, the property by which the cards were sorted changed. The number of perseverative errors- errors where the participant continued to sort cards by a property that was no longer correct- indicated executive function capacity such that a lower score indicated greater ability. This task is said to primarily measure cognitive flexibility- the ability to switch between or simultaneously hold multiple concepts in mind [30]. The paper and pencil Trail Making Test (TMT; [38]) was used as a second measure of executive function. This test measured time taken to connect written numbers in an ascending order (Trail A) compared to time taken to connect alternating numbers in ascending order and letters in alphabetical order (Trail B), for example, 1-A-2-B. Executive function was indicated by TMT derived- the time taken to complete Trial B minus Trial A, where a smaller difference score indicated greater executive function. This task is said to measure visual attention and task switching [41].

#### Habit

The self-report habit index [48] was used to measure the strength of participants’ unhealthy eating habits and sedentary behavior habits. For unhealthy eating, participants responded to the stem: “Eating an unhealthy diet is something…” and for sedentary behavior, participants responded to the stem: “Sedentary behaviors (such as TV viewing, internet browsing, lying down on the couch, sitting down and reading, driving instead of walking even for short distances) are something…”. For both unhealthy eating and sedentary behaviors, these stems were followed by the 12 items of the self-report habit index, for example: “…I do automatically”; “…I would find hard not to do”. Participants responded on 5-point Likert scales anchored by 1 = strongly disagree, and 5 = strongly agree. Both scales demonstrated excellent reliability at all three time points (unhealthy eating: α_T1_ = .959, α_T2_ = .963, α_T3_ = .964; sedentary behaviors: α_T1_ = .954; α_T2_ = .966; α_T3_ = .978).

### Procedure

The procedure has been described previously in Raman et al. [36]. In brief, participants were initially screened via phone for eligibility. Following this, a face-to-face session took place at which time executive function and habit outcomes were measured, and participants provided demographic and weight information. Participants then received three 90 min group-based behavioral weight loss therapy sessions over a period of 3 weeks. At the completion of the final session, participants were randomly allocated to CRT-O or no treatment. An external computer-based program was used to conceal randomisation and allocation to conditions. All assessment measures will be administered by the same clinical psychologist at baseline, after 7 to 9 weeks (3 weeks of behavioral weight loss and 4 to 6 weeks of either CRT-O or no treatment), and at the 3-month. However, data will be imputed and cleaned by an independent research assistant, and executive function will be measured by a computer. The RCT was registered with the Australian New Zealand Registry of Clinical Trials (trial id: ACTRN12613000537752).

### Data analysis

#### Main analyses

A series of 2 (condition: CRT-O, control) by 3 (time: baseline, post, follow-up) mixed ANOVAs were conducted on 1) WSCT performance, 2) TMT performance, 3) unhealthy eating habit strength, and 4) sedentary habit strength to determine the effect of CRT-O training on executive function and habit strength outcomes. Planned contrasts were conducted to test whether change in outcomes differed between the conditions from 1) baseline to post-intervention to determine the effect of the intervention, and from 2) baseline to follow-up to determine if effects were maintained.

#### Mediation analyses

To determine whether changes in habit strength outcomes were due to changes in executive function, mediation analyses were conducted using the PROCESS macro for SPSS [17] with 5000 bootstrap samples. These analyses were originally planned to be conducted using a composite executive function ability variable; however, scores on the WCST did not correlate with those on the TMT. Therefore, it was not appropriate to combine the two measures and they were analysed separately. Two parallel multiple mediation models were used to test the indirect effects of intervention on change in either unhealthy eating habit strength or sedentary behavior habit strength through change in both measures of executive function. Change in habit strength variables were created by subtracting follow-up scores from baseline scores, while change in executive function variables were created by subtracting post-intervention scores from baseline scores. Change from baseline to follow-up for habit outcomes was examined as habit theory would suggest that change in habit strength may be delayed or not discernible immediately after intervention. Change from baseline to post-intervention was examined for executive function as the intervention was hypothesised to influence executive function immediately. For each analysis, the significance of the indirect effects was assessed using 95% confidence intervals; calculated using 5000 bootstrap re-samples [17].

## Results

There were no differences at baseline between experimental (*n* = 42) and control (*n* = 38) conditions on any of the included outcome variables or by age or gender, all *ps* > .05. Further, there were no differences between those who remained in the study and those who dropped out (*n* = 17) on any outcome variable, age or gender, all *ps* > .05. However, participants were more likely to drop-out of the control condition (*n* = 12) than the experimental condition, *n* = 5; χ^2^ = 4.615, *p* = .032.

### Wisconsin card sort task

**Fig. 1:**
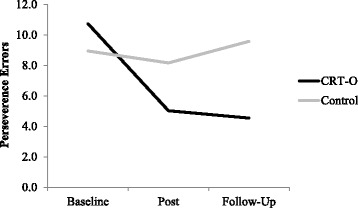
WCST performance at each time point separately for condition

There were main effects of time and condition which were qualified by a significant time by condition interaction, *F* (2,122) = 26.736, *p* < .001, Cohen’s *d* = 1.325. Contrasts examining these effects separately for each time comparison revealed that WCST perseverance errors reduced in the CRT-O condition, *MD* = − 5.854, *SD* = 4.216, but not the control group, *MD* = − 0.226, *SD* = 4.455, from baseline to post-intervention, *F* (1,61) = 22.928, *p* < .001, Cohen’s *d* = 1.086. Similarly, perseverance errors reduced in the CRT-O condition, *MD* = − 6.108, *SD* = 4.532, but not the control group, *MD* = 1.038, *SD* = 5.087, from baseline to follow-up, *F* (1,61) = 34.311, *p* < .001, Cohen’s *d* = 1.500, see Fig. 1.

### Trail making task

**Fig. 2:**
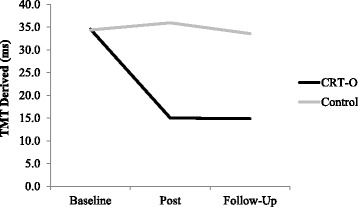
TMT performance at each time point separately for condition

There was a main effect of time which was qualified by a significant time by condition interaction, *F*(2,116) = 4.788, *p* = .010, Cohen’s *d* = 0.574. Contrasts examining these effects separately for each time comparison revealed that TMT performance improved in the CRT-O condition, *MD* = − 19.673, *SD* = 35.442, but not in the control group, *MD* = 0.870, *SD* = 24.858, from baseline to post-intervention, *F* (1,58) = 5.842, *p* = .019, Cohen’s *d* = 0.637. Similarly, TMT performance improved in the CRT-O condition, *MD* = − 19.791, *SD* = 37.945, but not the control group, *MD* = 0.467, *SD* = 32.538, from baseline to follow-up, *F* (1,58) = 4.869, *p* = .031, Cohen’s *d* = 0.578, see Fig. 2.

### Unhealthy eating habit

**Fig. 3:**
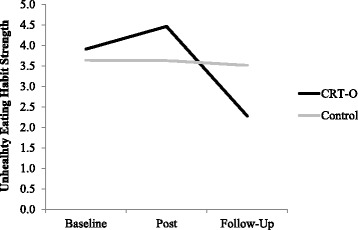
Unhealthy eating habit strength at each time point separately for condition

There were no significant main effects of time or condition, nor was the interaction between time and condition significant. However, planned contrasts- which analysed these effects separately for baseline to post-intervention and for baseline to follow-up- revealed that habit strength significantly reduced in the CRT-O group, *MD* = − 1.588, *SD* = 0.986, compared to the control group, *MD* = 0.032, *SD* = 1.021, from baseline to follow-up, *F* (1,60) = 39.101, *p* < .001, Cohen’s *d* = 1.616, but not from baseline to post-intervention, *F* (1,60) = .083, *p* = .778, Cohen’s *d* = 0.063, see Fig. 3.

### Sedentary behavior habit

**Fig. 4:**
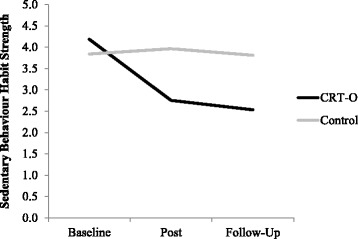
Sedentary behavior habit strength at each time point separately for condition

There were main effects of time and condition which were qualified by a significant time by condition interaction, *F* (2,120) = 30.770, *p* < .001, Cohen’s *d* = 1.432. Contrasts examining these effects separately for each time comparison revealed that sedentary habit strength reduced in the CRT-O condition, *MD* = − 1.412, *SD* = 0.946, but not in the control group, *MD* = 0.199, *SD* = 0.825, from baseline to post-intervention, *F* (1,60) = 65.326, *p* < .001, Cohen’s *d* = 2.086. Similarly, sedentary habit strength reduced in the CRT-O condition, *MD* = − 1.660, *SD* = 1.306, but not in the control group, *MD* = 0.170, *SD* = 0.591, from baseline to follow-up, *F* (1,60) = 42.949, *p* < .001, Cohen’s *d* = 1.692, see Fig. 4.

### Mediation analyses

**Fig. 5:**
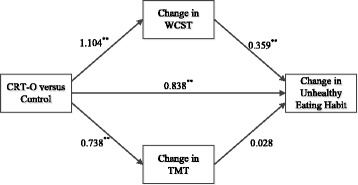
Multiple parallel mediation model demonstrating the indirect effect of condition on change in unhealthy eating habits through change in WCST performance and TMT performance. Standardised beta coefficients are shown. ^**^ Significant at .01 level. Total effect: β = 1.255, *p* < .01

The indirect effect of condition on change in unhealthy eating habit, through change in WCST perseverance errors^1^ was significant, β = 0.396, 95% [CI: 0.119, 0.790]. This indirect effect accounted for 31.56% of variance in the total effect and an additional 8.58% of the variance in unhealthy eating habits. The indirect effect of condition on change in unhealthy eating habit, through change in TMT performance was not significant, β = 0.026, 95% [CI: -0.108, 0.199], and only accounted for 1.62% of the variance in the total effect. The effect of intervention condition on change in habit strength was partially mediated by change in WCST performance, as the direct effect of condition on habit change remained significant once change in WCST was added to the model, β = 0.838, 95% [CI: 451, 1.687]. Overall, the direct and indirect effects accounted for 46.97% of the variance in change in unhealthy eating habits. See Fig. 5 for standardised coefficients between all variables in the model.

**Fig. 6:**
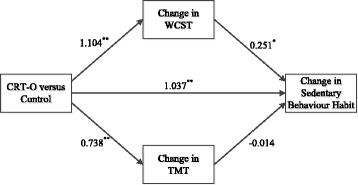
Multiple parallel mediation model demonstrating the indirect effect of condition on change in sedentary behavior habits through change in WCST performance and TMT performance. Standardised beta coefficients are shown. ^**^ Significant at .01 level, ^*^ significant at .05 level. Total effect: β = 1.304, *p* < .01

The indirect effect of condition on change in sedentary behavior habit, through change in WCST perseverance errors was significant, β = 0.277, 95% [CI: 0.033, 0.626]. This indirect effect accounted for 21.21% of variance in the total effect and an additional 3.91% of the variance in sedentary behavior habits. The indirect effect of condition on change in sedentary behavior habit, through change in TMT performance was not significant, β = − 0.010, 95% [CI: -0.140, 0.193], an only accounted for 0.8% of the variance in the total effect. The effect of intervention condition on change in habit strength was partially mediated by change in TMT performance, as the direct effect of condition on change in habit remained significant once change in TMT was added to the model, β = 1.037, 95% [CI: 0.758, 2.148]. Overall, the direct and indirect effects accounted for 44.95% of the variance in change in sedentary behavior habits. See Fig. 6 for standardised coefficients between all variables in the model.

## Discussion

This is the first study to examine the effects of CRT on habitual behavior, both sedentary activity and unhealthy eating, in individuals with obesity. Following from evidence that CRT improves executive function [26], we hypothesised that this improvement in executive function would provide individuals with the mental flexibility to break unwanted habits, which in turn would account for changes in lifestyle outcomes- specifically weight loss- as documented in a previous study (Raman et al., 2016). From the current analysis we found that CRT-O improved executive function, both WSCT and TMT, compared to the control group, at both post-treatment and three-month follow-up, with large effect sizes. Additionally, those in the CRT-O group also had reduced habitual sedentary behavior at post-treatment and follow-up, but only reduced habitual unhealthy eating at 3 month follow-up, with large effect sizes. Importantly, changes in habitual behavior were mediated by changes in one element of executive function that is, cognitive flexibility measured by performance on the WCST.

These findings are the first to demonstrate that improving executive function has a direct impact on habitual behaviors, building on previous research that has provided support for the effectiveness of habit based interventions in the promotion of weight loss [25]. Specifically, how enhancing self-regulatory skills and automaticity through habit-based intervention can lead to weight loss in adults. While previous research has shown the potential of executive function training to improve health outcomes in non-clinical populations [4], few studies have applied such training to individuals with obesity, or specifically examined the effect on unhealthy habits. Perseverance- as measured by the WCST- appeared to be the most influential element of executive function in changing unhealthy eating habits and sedentary behavior. This outcome is said to reflect cognitive flexibility [6], and has been associated with weight previously where it has been shown that individuals with anorexia nervosa or obesity have more rigid thinking styles and difficulty perceiving alternative ways of coping with problems compared to healthy weight controls [10]. In the current context, it appeared that improving this element of executive function may have allowed participants to break their unhealthy routines.

The TMT is said to measure different elements of executive function compared to the WCST; namely, visual attention and task switching [41]. This may partially explain why improvements on the TMT did not mediate changes in habit strength in the same way that changes in WCST performance did. These measures did not correlate, providing further evidence that these tasks index distinct elements of executive function. It appears that TMT performance is not relevant to interrupting the unhealthy eating and sedentary behavior habits of individuals with obesity. While previous research has demonstrated TMT deficits in individuals with anorexia nervosa compared to healthy weight controls [39], research examining differences between individuals with obesity and healthy weight controls has not consistently found this effect [11]. Thus, future research that examines the role of TMT performance or other measures of task switching and visual attention in individuals with obesity is warranted.

The finding that changes in executive function mediated changes in unhealthy habits provides a mechanistic explanation for how habits are broken (or at least reduced).

Habits are primarily interrupted when the behavior becomes impossible (i.e., unhealthy menu option removed) or the context in which the cue to act changes or the value of the outcome changes [22]. One interpretation of the results is that individuals with greater cognitive flexibility may be able to use these interruptions to adjust their behavior in line with their weight loss goals rather than persisting with an unhealthy choice. Interestingly, habit strength for unhealthy eating habits did not improve from baseline to post-intervention, while sedentary habits did. The greater lag between intervention and effect for this outcome may indicate that unhealthy eating habits are more difficult to break. The results also reflect a qualitative difference between the two behaviors- stopping an unhealthy eating habit versus engaging in more physical activity. Previous research has demonstrated a distinction between health behaviors that require the inhibition or initiation of a response in terms of associated processes [29, 35].

### Limitations

Executive function encompasses a broad set of processes [24]. In the current study, only two elements of executive function were examined. It is possible the CRT-O also improved other elements of executive function and that these are just as important- and therefore also useful targets for intervention- as cognitive flexibility. Additionally, the follow-up period was relatively short. It is possible that these effects may not persist beyond 3 months. This is important to consider when interpreting changes in habit outcomes, which may eventually return to baseline if the habit is reinstated. Additionally, there is evidence for the bi-directionality of the relationship between executive function and weight [42]. While executive function may have improved immediately as a result of CRT-O, the maintenance of these effects at 3 month follow-up may have been facilitated by the reduction in weight. A further limitation was the self-report habit index measure that was used. This used a general measure of unhealthy eating and sedentary behavior rather than specifics around, for example, avoiding saturated fat and increasing fruit and vegetable consumption. Research has shown that these behaviors may have different drivers [3]. The conclusions drawn in this manuscript are also limited by the fact that the analyses were conducted post-hoc and future research is needed to replicate these findings in additional studies. Additionally, given that this was a post-hoc analysis, we were constrained to certain analyses that the sample size would allow. Given more power, it would have been useful to test a larger model whereby weight loss itself was included as a variable.

## Conclusions

CRT-O appeared to improve specific elements of executive function. Changes in executive function mediated changes in unhealthy eating habits and sedentary behavior habits. These results are important to consider when implementing behavioral weight loss programs to individuals with obesity. Specifically, interventions that include components that train executive function may be particularly useful as improvements in executive function relate to reduced unhealthy eating and sedentary behavior habits, which have flow on effects to weight loss. Furthermore, these results have implications for habit theory suggesting that cognitive flexibility is a key mechanism in the reduction of unwanted habits. Moreover, cognitive flexibility may broadly reflect individual differences in the ability to break habitual behavior.

## Abbreviations

BMI: Body mass index
CI: Confidence interval
CRT: Cognitive remediation therapy
CRT-O: Cognitive remediation therapy – obesity
MD: Mean difference
SD: Standard deviation
TMT: Trail Making Test
WCST: Wisconsin Card Sorting Test

## Footnotes

1 Note that change in WCST perseverance errors and change in TMT performance variables are calculated as the difference between baseline and post-intervention scores as the intervention was hypothesized to influence this ability immediately, whereas change in unhealthy eating habit and change in unhealthy sedentary behaviour are calculated as the difference between baseline and follow-up scores as habit theory would suggest that change in habit strength may be delayed or not discernible immediately after intervention. Analysis revealed that changes in WCST and TMT observed from baseline to post-intervention were maintained from post-intervention to follow-up, and examining change from baseline to follow-up did not alter the results.
